# Description and Functional Benefits of Meeting Frequency, Intensity, and Time of Resistance and Cardiovascular Exercises: A Study of Older Adults in a Community-Based, Slow-Stream Rehabilitation, Hospital-to-Home Transition Program

**DOI:** 10.1177/23337214221096303

**Published:** 2022-05-20

**Authors:** Melody Maximos, Paul Stratford, Ada Tang, Vanina Dal Bello-Haas

**Affiliations:** 1 School of Rehabilitation Science, Faculty of Health Sciences, 3710 McMaster University, Hamilton, ON, USA

**Keywords:** older adults, cardiovascular exercise, resistance exercise, function, slow-stream rehabilitation

## Abstract

This prospective cohort study described cardiovascular and resistance exercises completed by older adults in a community-based, slow-stream rehabilitation, hospital-to-home transition program; compared exercises completed to the American College of Sports Medicine (ACSM) exercise guidelines; and, assessed differences in Late Life Function and Disability Index (LLFDI)-Function Component (FC) between older adults who met and did not meet the ACSM guidelines. Descriptive statistics and Factorial ANCOVA were conducted. For cardiovascular exercise 59.3% of participants met frequency, 73.4% met intensity, and 35.9% met time. For resistance exercise, 67.2% of participants met frequency, 42.2% met intensity, and 76.6% number of repetitions. Participants who met both frequency and time for cardiovascular exercise had higher LLFDI-FC scores, as did those who met intensity and/or number of repetitions for resistance exercise. The findings provide support that older adults engaged in a slow-stream rehabilitation program can meet the ACSM exercise guidelines for community-dwelling older adults, and that meeting the guidelines improves function.

Approximately 30%–60% of older adults experience difficulties completing activities of daily living (ADL), and have an increased risk of falls, hospital readmission and institutionalization post-hospital discharge (Covinsky et al., 2003; Kortebein, 2009). Research has found that one third of older adults have not recovered to their pre-admission status 1 year after hospitalization (Paolucci et al., 2001; Zisberg et al., 2015). These challenges are thought to be related to rehabilitation goals not being met prior to discharge, reduced mobility, and decrease in muscle mass experienced during hospitalization (Cress et al., 2006; Rimmer, 2005). Exercise interventions have been shown to substantially improve older adults’ ability to maintain or return to pre-admission function following hospitalization (Courtney et al., 2012; Theou et al., 2011). For gains to be made however, the exercise intervention must be physiologically adequate to cause gains in muscle strength and endurance and be matched to the older adult’s abilities and goals (White et al., 2015). Findings from two systematic reviews examining the effects of exercise interventions found that interventions aimed at improving strength, endurance and balance have been associated with improved mobility, ability to complete activities of daily living (ADLs) without assistance, and quality of life compared to usual care in institutionalized care, and community and hospital rehabilitation settings (de Labra et al., 2015; Theou et al., 2011). Despite the documented benefits of exercise for older adults, exercise intensity, time and frequency tends to be under-prescribed or inconsistently prescribed (White et al., 2015).

A model of care available for older adults with complex health conditions, including frailty and severe injury, who are transitioning from hospital-to-home is slow-stream rehabilitation (SSR). SSR is longer in program length with shorter individual sessions at lower intensity (Maximos et al., 2019). While SSR programs are intended to meet rehabilitation needs of older adults and are beneficial for increasing function and decreasing institutionalization, little has been documented about specific SSR exercise interventions (Maximos et al., 2019). Despite programs being referred to as SSR in the literature, exercise interventions vary and no clear exercise parameters have been described or published (Maximos et al., 2019), and there is a lack of research that has focused on exercise guidelines. Descriptions of exercise parameters and development of SSR exercise guidelines would decrease the heterogeneity of SSR exercise interventions e.g., type of exercises, and duration, intensity, and frequency of exercises (Maximos et al., 2019); and, this information would guide the implementation of guidelines that meet the needs and goals of the older adults in these types of programs.

Currently, exercise guidelines that exist for community-dwelling older adults (American College of Sports Medicine, 2017, p. 188) and older adults with frailty (Mols Bayles et al., 2009) may be applicable to older adults in SSR programs; refer to Table 1 for more information and a comparison. Community-dwelling older adult exercise guidelines are intended for older adults aged 65 years and older and provide specific details for frequency, intensity and time for cardiovascular and resistance exercise (American College of Sports Medicine, 2017, p. 188). Frailty exercise guidelines are intended for older adults with decreased physiological reserve and multisystem dysregulation. Compared to non-frail older adults, frail older adults are more dependent and recover more slowly from illness (Mols Bayles et al., 2009). The frailty exercise guidelines suggest a multicomponent exercise program with a frequency of at least a three times a week minimum for both cardiovascular and resistance exercise(Mols Bayles et al., 2009). However, frailty exercise guidelines do not specify intensity for cardiovascular or resistance exercises or the number of resistance exercises or repetitions for resistance exercises, and provide a wide range for cardiovascular exercise time, from five to 60 minutes.

**Table 1.** Frequency, Intensity, and Time Parameters for Exercise Type: Comparison of Exercise Guidelines.

Exercise Type	FIT parameter	Community-dwelling older adult (American College of Sports Medicine, 2017 pp.188 pp.188)	Frailty (Mols Bayles et al., 2009)
Cardiovascular	Frequency	3–5 days/week	3–5 days/week
	Intensity	Moderate to vigorous intensity^a^	Not described
	Time	20–60 minutes	5–60 minutes
Resistance	Frequency	At least 2 days/week	3 days/week
	Intensity	60–80% 1-RM^b^	Not described
	Time/repetitions	1-3 sets of 8–12 repetitions for each exercise	20 minutes

The CR-10 Borg Scale ® (Borg, 1990) was used to measure intensity in the study.

*Note.* 1-RM = one repetition maximum; FIT = Frequency, Intensity, Time.

^a^For cardiovascular exercise, moderate intensity is equivalent to a 3 to 4 on the CR-10 Borg Scale ® (Borg, 1990) and vigorous intensity is equivalent to 7 to 8, CR-10 Borg Scale ® (Borg, 1990).

^b^For resistance exercise, 60–80% of one repetition max (intensity) is equivalent to 5 to 8 on CR-10 Borg Scale ® (Morishita et al., 2019).

Having clear and appropriate frequency, intensity and time guidelines for each exercise type is important to ensuring exercises are being completed at a level that will physiologically lead to functional gains (White et al., 2015). The overarching aim of this study was to contextualize frequency, intensity, time and type (FITT) parameters of exercises completed by older adults engaged in a community-based SSR program and explore how meeting exercise guidelines in a community-based, SSR programs has the potential to improve functional outcomes for older adults.

## Study Objectives

The primary purpose of this study was to describe the frequency, intensity, type, and time (FITT) parameters for exercises completed by older adult participants in a community-based SSR, hospital-to-home transition program; and to compare the FITT parameters of completed exercises to established exercise guidelines for community-dwelling older adults (American College of Sports Medicine, 2017).

The second purpose was to explore whether there was a difference in function, as measured by the Late Life Function and Disability Index-Function Component, between older adult participants in a community-based, SSR, hospital-to-home transition program who met American College of Sports Medicine (ACSM) cardiovascular and resistance frequency, intensity, time/number of repetition exercise guidelines for community-dwelling older adults compared to those who did not meet the guidelines.

## Methods

### Study Setting

The community-based, SSR hospital-to-home transition program is located in one of the 14 Local Health Integration Networks in the province of Ontario. The aim of the program is to assist community-living older adults transitioning from hospital-to-home through the provision of nursing, physiotherapy, recreation therapy, nutrition, and support services as needed. Older adult participants were eligible to participate in the program if they were referred to the program by a regulated healthcare professional, were medically stable and able to safely live at home with or without supports. Participants attended the program 5 days a week for 1 month from 9:00a.m. to 3:00 p.m., with transportation and lunch provided. Participants typically engaged in individual exercise programs, cognitive and social activities, and education sessions.

#### SSR Community-based Hospital-to-Home Exercise Program

Older adults’ medical history was reviewed upon entry into SSR program. Precautions, relative and absolute contraindications, risks related to participation in the program, and the need for any additional medical follow-up were identified. Older adults’ exercise program was prescribed by the physical therapist and monitored by an assistant who provided support to ensure safety, aided with transfers on and off the exercise equipment and logging of completed exercises. Exercises prescribed included: one to two cardiovascular and three to five resistance exercises, three to 5 days a week. Duration, number of repetitions and intensity were as tolerated. Prescribed balance and flexibility exercises, tailored to individual needs, were completed at home. When older adults began the program, they were provided with a logbook that listed various types of exercises and the older adult’s individual prescription was highlighted. The older adult recorded the date, exercises completed, number of repetitions, and amount of time for cardiovascular exercise.

#### Study Design and Participants

This study was a prospective cohort study of male and female adults 60 years of age and older who were recently discharged from the hospital and taking part in a 4-week SSR program. This study was approved by the Research Ethics Board and participants provided written informed consent. At baseline, a demographic questionnaire, Montreal Cognitive Assessment (MOCA) and Late Life Function and Disability Index (LLFDI)- Function Components were completed. The MOCA is a 30-question test that evaluates seven domains of cognitive ability (Nasreddine et al., 2005). Scores range from 0 to 30 and a cut-off score of less than 26 indicates cognitive impairment (Nasreddine et al., 2005). During the program a research assistant observed the older adults exercise program and asked them to rate their intensity. At discharge the LLFDI- Function Component was administered.

#### Late Life Function and Disability Index

The LLFDI- Function Component was administered by a research assistant at baseline and discharge (4-week point) from the program. The LLFDI is a patient reported outcome measure that has two distinct domains: a Disability Component and a Function Component (Jette et al., 2002). For this study, we examined the Function Component only because we were interested in determining whether meeting exercise guidelines made a difference in older adult participants’ ability to complete functional tasks. The Function Component assesses functional limitation, defined as the difficulty an older adult individual experiences completing discrete actions or activities, such as putting on and taking off a coat, or going up and down a flight of stairs using a handrail (Jette et al., 2002). The LLFDI-Function Component consists of 32 questions that ask about: basic lower extremity function, advanced lower extremity function, and upper extremity function (Jette et al., 2002). LLFDI-Function Component scores range from 0 and 100 and lower scores indicate greater difficulty in performing physical functional tasks (Jette et al., 2002; Sayers et al., 2004). For the LLFDI-Function Component, the minimal clinically important difference (MCID) for small change is 2 (Beauchamp et al., 2019). The LLFDI-Function Component in community-dwelling older adult populations had good validity and test-retest reliability (Beauchamp et al., 2014).

#### Frequency, Intensity, Type and Time (FITT) Parameters Data Collection

Participants were observed during their exercise program by the research assistant. The research assistant recorded the types of exercises completed, the amount of time for each exercise, and the number of times exercises completed per week (frequency). At end of program, participant logbooks and data collected by the research assistant were reconciled for cross-checking purposes and inputting. For cardiovascular exercise, time was measured as duration of time spent engaging in cardiovascular exercise. All cardiovascular exercises were completed using either a NuStep® recumbent cross trainer, an arm cycle ergometer, or both. For resistance exercise, time was measured as the number of repetitions completed for each muscle group. All upper and lower body resistance exercises were completed using strength training equipment in a seated position. Participants were asked by a research assistant to rate their perceived exertion (intensity) using the CR-10 Borg Scale ® Rate of Perceived Exertion (RPE) (Borg, 1990). The CR-10 Borg Scale® was administered to each participant during one upper body resistance exercise, one lower body resistance exercise, and a cardiovascular exercise for the duration of their program. Flexibility and balance exercises prescribed as part of a home exercise program were not included as part of the study.

#### CR-10 Borg Scale® Rate of Perceived Exertion (RPE)

The CR-10 Borg Scale® is a 11-point, category-ratio scale that measures perceived exertion (Borg, 1982; the scale with correct instructions can be obtained from Borg Perception, see the home page: www.borgperception.se). The CR-10 Borg Scale® is broken down into five verbal descriptors of perceived exertion: 0–2 = *weak*, 3–4 = *moderate*, 5–6 = *strong or heavy*, 7–8 = *very strong or heavy;* and, 9–10 = extremely *strong or maximal* (Buckley & Borg, 2011). The CR-10 Borg Scale® has been used to establish safe levels of exercise and parallel physiological variables for community-dwelling older adults (Eston & Thompson, 1997). Previous studies have shown that the CR-10 Borg Scale® is a valid and useful measure for measuring exercise intensity for older adults with a variety of chronic conditions engaging in cardiovascular and resistance exercise (Morishita et al., 2019; Donath et al., 2013) and, correlates with heart rate during cardiovascular exercise (Donath et al., 2013) and repetition maximum (RM) for resistance exercise in older adults (Buckley & Borg, 2011; Morishita et al., 2019)).

#### Exercise Guidelines

The ACSM exercise guidelines for community-dwelling older adults were chosen for this study because they: 1) were the most applicable to our program population (e.g., community-dwelling, older adult participants); and 2) provide specific details regarding frequency, intensity and time parameters for both cardiovascular and resistance exercises (American College of Sports Medicine, 2017, p. 188). Refer to Table 1 for the ACSM guidelines for community dwelling older adults. For cardiovascular exercise moderate intensity was considered between a 3 to 4, CR-10 Borg Scale® and vigorous intensity was a 7 to 8, CR-10 Borg Scale® (Borg, 1990). According to literature assessing the CR-10 Borg Scale® use for resistance exercise in older adults, 60–80% of 1-RM is equivalent to 5 to 8 on CR-10 Borg Scale® (Morishita et al., 2019).

### Analysis

All statistical analyses were completed using Stata 14.0, with a *p*-value for significance set to <0.05. Means, standard deviations, medians for variables that were not normally distributed, and minimum and maximum values were calculated for continuous variables; and, frequencies were calculated for nominal variables. Sample size was based on convenience and no priori sample size for analysis was calculated.

#### Primary Objective

To address the primary purpose, descriptive statistics were calculated. To examine distribution of the data, percentages, minimum and maximum values, mean, median and mode were calculated for intensity, frequency, and duration/repetitions of cardiovascular and resistance exercise. To compare ACSM guidelines and the exercises completed by the older adult participants during their exercise sessions, the percentage of older adult participants who met and who did not meet the guidelines for frequency, intensity and time parameters for cardiovascular and resistance exercises were calculated.

#### Secondary Objective

For cardiovascular exercise, a Factorial ANCOVA was conducted to determine whether there was a statistically significant difference for function, as measured by the LLFDI- Function Component (dependent variable), for the following independent factors: cardiovascular exercise frequency (met/not met), time (met/not met), intensity (met/not met) and their interactions; with age and baseline LLFDI-Function Component score as covariates. Minimum met criteria for cardiovascular exercise were defined as follows: frequency – three times a week, intensity – moderate (3–4, CR-10 Borg Scale®); time – 20 minutes (American College of Sports Medicine, 2017, p. 193). Only interactions with five or more participants in the cell were conducted.

For resistance exercise, a Factorial ANCOVA was conducted to determine whether there was a statistically significant difference for function, as measured by the LLFDI-Function Component (dependent variable), for the following independent factors: resistance exercise frequency (met/not met), time (met/not met), intensity (met/not met) and their interactions; with age and baseline LLFDI-Function Component score as covariates. Minimum met criteria for resistance exercise were defined as follows: frequency – two or more times a week; intensity – strong (5, CR-10 Borg Scale®); time – five repetitions (American College of Sports Medicine, 2017, p. 193). Only interactions with five or more participants in the cell were conducted.

## Results

### Descriptive Statistics

#### Participants

A total of 64 participants completed the community-based, SSR program during the study timeframe. The mean age was 78.4 years (SD= 9.8) and 62.5% were female. The mean number of chronic conditions was 3.2 (SD= 2.0). The mean MOCA score was 21.8 (SD= 5.21), indicating mild cognitive impairment (Nasreddine et al., 2005). The mean baseline LLFDI-Function Component score was 44.5 (SD = 9.7), and the mean discharge LLFDI-Function Component score was 46.2 (SD = 6.5), indicating that participants had severe limitations or difficulties completing physical activities (Haley et al., 2002). Refer to Table 2 for participant demographics.

**Table 2.** Demographics of participants that completed the Community-Based, Slow Stream Rehabilitation, Hospital-to-Home Transition Program.

**Characteristic**		**Mean (SD)/ Percentage (N=64)**
Age		78.4 (9.8) years
Sex	Male	37.5%
	Female	63.5%
Educational Level	Grade School	42.2%
	High School	25%
	College	18.8%
	University	14%
Living Situation	Alone	48.4%
	With Spouse	31.2%
	With Child	20.4%
Living Location	House/Townhouse	45.3%
	Apartment	42.2%
	Other	12.5%
Assistive Devices	None	1.6%
	Cane	6.3%
	Walker	82.8%
	Wheelchair	9.4%
Number of Medications		3.2 (2.0)
Number of Conditions		5.8 (2.6)
MOCA score		21.8 (5.2)
Baseline LLFDI-Function Component score		44.5 (9.7)
Discharge LLFDI-Function Component score		46.2 (6.5)

^*^LLFDI= Late Life Function and Disability Index, MOCA= Montreal Cognitive Assessment.

#### Types of Exercise Completed

Cardiovascular exercise was completed by all participants, with 59 (92.2%) of participants completing one cardiovascular exercise, while 5 (7.8%) completed more than one cardiovascular exercise per exercise session. Lower body resistance exercises were completed by 56 (87.5%) participants. Participants 0–2 lower body resistance exercises, specifically the following muscle group exercises: hamstring and quadriceps. Upper body resistance exercises were completed by 52 (81.3%) participants. Participants completed 0–3 upper body resistance exercises. Muscle group exercises included: pectoral, deltoid, and triceps.

#### Frequency of Prescribed Exercises

Participants completed cardiovascular exercise a median of 3.2 days/week, lower body resistance exercise a median of 3.2 days/week, and upper body resistance exercise a median of 2 days/week.

#### Intensity of Exercises

Median RPE rating for: cardiovascular exercise was 4.2 -*somewhat hard* (CR-10 Borg Scale ® RPE verbal descriptor, Borg, 1990), 5.5 – *strong or heavy* (CR-10 Borg Scale ® RPE verbal descriptors, Borg, 1990) for lower body resistance exercise, and 4.7 – *moderate to strong* (CR-10 Borg Scale ® RPE verbal descriptor, Borg, 1990) for upper body resistance exercises.

#### Time of Exercises

Participants engaged in a median of 14.2 minutes of cardiovascular exercise per session. For both upper and lower body resistance exercises, the median number of repetitions completed was 20. Refer to Supplement Table 1A for the FITT parameters of completed exercises.

### Comparison of Exercises Completed by Older Adult Participants to the American College of Sports Medicine Exercise Guidelines

#### Cardiovascular Exercise

Thirty-eight (59.4%) met the guidelines for frequency. Forty-seven (73.4%) met the guidelines for intensity. Twenty-three (35.9%) met the guidelines for time. Twenty-nine (45.3%) met frequency and intensity guidelines. Twenty-six (40.6%) met frequency and time guidelines. Thirty-five (54.7%) met intensity and time guidelines. Twenty-one (32.8%) met frequency, intensity, and time guidelines for cardiovascular exercise.

#### Resistance Exercise

Forty-three (67.2%) met guidelines for frequency. Twenty-seven (42.2%) met guidelines for intensity. Forty-nine (76.6%) met guidelines for number of repetitions. None of the participants completed resistance exercises for eight to 10 muscle groups. Twenty-eight (43.7%) met frequency and intensity guidelines. Thirty-four (53.1%) met frequency and number of repetition guidelines. Twenty-five (39.1%) met intensity and repetition guidelines. Nineteen (29.7%) met frequency, intensity, and number repetitions guidelines.

### Difference in LLFDI-Function Component Score Between Those Who met and did not Meet FIT Guidelines for Cardiovascular and Resistance Exercise

The Factorial ANCOVA with LLFDI-Function Component score as the dependent variable; cardiovascular exercise frequency (met/not met), time (met/not met), intensity (met/not met) as the independent factors; age and baseline LLFDI-Function Component score as covariates. Interaction effect for frequency and intensity, and the three-way interaction between intensity, frequency and times were not calculated due to small number of participants in each cell. Baseline LLFDI-Function Component was a significant covariate in the model (F_(1,56)_ = 117, *p* < .001, *n*_*p*_^*2*^ *= 0.67*). Those who had higher LLFDI-Function Component baselines scores also had higher LLFDI-Function Component discharge scores. There were no main effects, however there was a significant interaction effect between frequency and time (F_(1,56)_ = 8.4, *p* = .005, *n*_*p*_^*2*^ *= .13*) . A post-hoc with Sidak multiple comparison analysis showed that older adult participants who met the ACSM guidelines for both frequency and time had statistically greater LLFDI-Function Component discharge scores (F_(2,56)_ = 4.60, *p* = 0.01, $\bar{X}\;$ = 48.3, CI = 46.9, 49.7), compared to participants who met time guidelines alone ( $\bar{X\;}$ = 43.9, *p* <.001, CI = 41.6, 46.2) or met frequency guidelines alone ( $\bar{X}$ = 44.4, *p* < .001, CI = 46.9, 49.7). Refer to Table 3.

**Table 3.** Factorial ANCOVA for Late Life Function and Disability Index - Function Component Discharge Score and Independent Variables: Cardiovascular Exercise Guidelines Frequency, Intensity and Time (met/not met).

Source^a^	Sum of Squares	df	Mean Square	F Value	*p* Value
Age	26.4	1	26.4	2.1	.15
Baseline late life function and disability index -Function component	1499	1	1499	117	<.001**
Frequency	8.9	1	8.9	0.7	.41
Intensity	18.3	1	18.3	1.4	.24
Time	0.11	1	0.11	0.01	.92
Frequency and time interaction	108.2	1	108.2	8.4	.005**
Time and intensity interaction	33.3	1	33	2.6	.11
Error	717.5	55	12.8		
Total	2620.8	62	41.6		

*Note.* ** *p*<0.01

^a^Interaction effect for frequency and intensity and three-way interaction between intensity, frequency and time was not calculated due to number of participants in interaction groups.

The Factorial ANCOVA with LLFDI-Function Component score as the dependent variable; resistance exercise frequency (met/not met), time (met/not met), intensity (met/not met) as the independent variables; age and baseline LLFDI-Function Component score as covariates. Interaction effect for a three-way interaction between intensity, frequency and repetitions was not calculated due to the small number of participants in each cell. The Factorial ANCOVA found that baseline LLFDI- Function Component was a significant covariate in the model (F_(1,55)_ = 114.4, *p* < .001, *n*_*p*_^*2*^ *= 0.67*). Those who had higher LLFDI-Function Component baselines scores also had higher LLFDI-Function Component discharge scores. There were no main effects, however there was a significant interaction effect between intensity and number of repetitions (F_(1,55)_ = 6.05, *p* = .017, *n*_*p*_^*2*^*= 0.10*). Post-hoc with Sidak multiple comparison analysis showed that older adult participants who met the ACSM guidelines for either intensity ( $\bar{X}$ = 47.2, *p* < .001, CI = 45.5, 48.9) or repetitions ( $\bar{X}\;$ = 47.9, *p*< .001, CI = 45.7, 50.2), or both ( $\bar{X}\;$ = 46.6, *p* < .001, CI = 44.2, 47.9) had higher LLFDI-Function Component discharge score in comparison to not meeting either $(\bar{X}$ = 43.5, *p* < .001, CI = 41.2 45.8). Refer to Table 4.

**Table 4.** Factorial ANCOVA for Late Life Function and Disability Index-Function Component Discharge Score and Independent Variables: Resistance Exercise Guidelines Frequency, Intensity and Time (met/not met).

Source^a^	Sum of Squares	df	Mean Square	F Value	*p* Value
Age	22.8	1	22.8	1.7	.19
Baseline late life function and disability index total function score	1527.6	1	1527.6	114.4	<.001**
Frequency	31.4	1	31.4	2.3	.13
Intensity	9.6	1	9.6	0.7	.39
Number of repetitions (time)	11	1	11	0.8	.37
Frequency and intensity interaction	3.7	1	3.7	0.3	.66
Frequency and repetitions (time) interaction	25.5	1	25.5	1.9	.17
Intensity and number of repetition (time) interaction	80.8	1	80.8	6.1	.017*
Error	734.7	55	13.4		
Total	2620.8	62	41.6		

*Note.* ** *p*<0.01, * *p* < 0.01

^a^Interaction effect for three-way interaction between intensity, frequency and repetitions was not calculated due to number of participants in interaction groups

## Discussion

To be beneficial to older adults transitioning from hospital-to-home, exercise interventions must address the complex needs of this population and align with relevant exercise recommendations and contraindications accordingly (Guthrie et al., 2012). With the paucity of literature available on specific exercise components in SSR programs (Maximos et al., 2019), the results of this study begin to contextualize FITT exercise parameters and how they may relate to functional gains in older adults recently discharged from hospital. The demographics (age, number of chronic conditions) and functional status (cognitive impairment, ADL performance) of participants in this study engaged in community-based SSR are similar to those of older adults completing hospital-based inpatient SSR programs– e.g., mean age range of 72–82 years, 47–81.5% female, and the majority had multiple comorbidities and mild cognitive impairment (Maximos et al., 2019). These similarities support the notion that older adults with complex healthcare needs requiring SSR can effectively complete their program in the community rather than in institutionalized settings.

Previous research of exercise-based SSR interventions did not report the specifics of the rehabilitation sessions making comparisons of any exercises difficult (Maximos et al., 2019). Data from inpatient SSR programs show that participants engaged in 30- to 60-minute sessions two to five times a week (Maximos et al., 2019). Yet, the type of exercises, time of each individual exercise and intensity was not reported and no guidelines for exercise program design was used or discussed within the reported literature. In this study we found that more than half of the participants in the current study completed cardiovascular and resistance exercises at the recommended frequency, met the intensity guidelines for cardiovascular exercise and met the repetition (time) guidelines for resistance exercise.

Given the complex presentation of conditions and deficits, our participants could be considered frail according to Rockwood & Mitnitski 's (2007) characterization of frailty as deficit (symptoms, signs, disease, disability) accumulation. Yet, specific FITT parameters are lacking in frailty exercise guidelines (Mols Bayles et al., 2009). Systematic reviews describing exercise interventions for frail older adults found that most resistance and cardiovascular exercise programs were conducted between two to three times per week (Cadore et al., 2013; de Labra et al., 2015; Theou et al., 2011). Resistance exercise intensity ranged from 30%-80% of 1-RM, yet number of repetition were consistent between 8–12 repetitions with a range of one to three sets (Cadore et al., 2013; de Labra et al., 2015; Theou et al., 2011). Intensity and duration for cardiovascular exercise were not clearly described in two (de Labra et al., 2015; Theou et al., 2011) of the three systematic reviews due to the variation of the interventions. One of the systematic reviews did suggest cardiovascular exercise be done at an intensity of 3–4 RPE starting at five to 10 minutes and increasing to 15–30 minutes (Cadore et al., 2013). The lack of clear guidelines, lack of description of exercise parameters and differences as to whether older adults with complex healthcare needs or who are frail should engage in exercise at higher intensities, for longer durations or at greater frequencies result in variability in interpretation of recommendations and in designing and prescribing an effective exercise intervention or program. This may increase the risk of under prescription of exercise FIT parameters and may result in parameters not aligning with the physiological ability of older adults (White et al., 2015), leading to decreased benefit and difficulty returning to independent community living post-hospitalization (Guthrie et al., 2012).

Almost 75% of participants in our study were able to engage in cardiovascular exercise at intensities higher than that recommended by the most recent systematic review published by Cadore et al. (2013), and approximately 60% exercised three or more times a week. However, only one-third were able to meet the time parameter for cardiovascular exercise, and none completed the recommended eight to 10 resistance exercises. These findings may not have been solely due to the older adults’ physical capacity to exercise but rather due to the program structure and allotted time in the gym. Therefore, it may be possible that the participants are able to meet the guidelines for both cardiovascular time and number of resistance exercises.

Participants who met both the ACSM frequency and time guidelines for community-dwelling older adults had statistically higher LLFDI-Function Component scores, however intensity did not have a significant influence on function scores. This suggests that it is the total amount of time of cardiovascular exercise completed over the week that is important. The ACSM guidelines indicate that cardiovascular exercise time can be accumulated over the course of the day but should be completed in bouts of 10 minutes minimum for physiological gains for a total of 60–300 minutes over a week (American College of Sports Medicine, 2017, p. 188). A systematic review looking at the benefits of high versus moderate intensity aerobic exercise found mixed results – eight studies reported similar benefits in physical outcomes between the two exercise groups, and seven reported greater improvement in physical outcomes with higher intensity (Keating et al., 2020). It may be that intensity is not as critical for changes in function, but rather intensity is more important for changes in cardiovascular parameters e.g., VO_2_ max. In order to sustain benefits in function, a review by Frankel et al. (2006) suggests increasing duration of exercise before intensity and ensuring duration is matched to the individual’s ability is important.

In our study, participants who met the ACSM guidelines for either intensity or repetitions or both for resistance exercise had higher scores for LLFDI-Function Component at discharge compared to those that did not meet either parameter. Literature assessing intensity and repetitions for resistance exercise has found that physical benefits are dependent on both parameters. Fiatarone et al. (1994) was one of the first to show that high intensity resistance exercises are feasible and effective in improving strength and gait velocity for frail institutionalized older adults. High intensity (80% 1-RM) resistance exercise for frail older adults was more effective at producing gains in physiological and functional outcomes compared to low intensity (40% 1-RM) resistance exercise, while maintaining the same number of repetitions (Seynnes et al., 2004). However, in the study by Vincent et al. (2002), groups either completed eight repetitions at 80% 1-RM or 13 repetitions at 50% 1-RM and both had similar improvements in strength, endurance and stair climbing ability, suggesting that there does not appear to be a difference in physical performance as long as adjustments for the number of repetitions was made (Vincent et al., 2002). Therefore, if high-intensity is preferred by and safe for the older adult participant, there is research to support this as an effective and well-tolerated method of resistance exercise for older individuals with complex healthcare needs (Valenzuela, 2012). In contrast, if the older adult prefers lower intensity or there are safety concerns, then a higher number of repetitions at a lower intensity should be completed, and benefits can still be realized (Valenzuela, 2012).

### Limitations

This study was observational study that represents a ‘snapshot in time’ of an exercise program completed by older adults while participating in a short duration, hospital-to-home SSR program. It is possible that we would have seen greater increases in LLFDI scores if the exercise program was of longer duration.

Program closures due to influenza outbreaks, changes in staffing, and participant dropout from the SSR program not related to exercise were beyond the control of researchers and affected data collection and sample size. Furthermore, since the analysis was based upon observational data and not a randomized control trial with a priori sample size calculation, the number of participants available for each cell of the Factorial ANCOVA was not controlled. To gain a better understanding of the interaction between frequency, intensity and time guidelines, a randomized study would be required. Flexibility and balance exercise recommendation are also part of the ACSM guidelines community-dwelling older adults but were not examined in this study.

### Implication and Future Direction

Findings regarding cardiovascular exercise that older adults could be given the choice between engaging in 20 or more minutes of cardiovascular exercise at one time or engaging in smaller bouts of exercise e.g., five to 10 minutes throughout the day. Similarly, in considering resistance exercise prescription, the total amount of resistance was found to be important and could be achieved in various ways while considering the older adult’s needs and preference. Thus, older adult participants should be encouraged to work towards their ability rather than perceived limits using established guidelines.

Additional studies are needed to further develop optimal exercise guidelines for older adults with complex healthcare needs who are transitioning from hospital-to-home to guide clinicians. Studies, with a priori sample size calculation, should be conducted as a next step. Future studies should also examine balance and flexibility exercise guidelines to gain more understanding of the effect of meeting these exercise type parameters on functional status in older adults recently discharged from hospital.

## Conclusion

This study is the first to contextualize FIT of cardiovascular and resistance exercise and how they may relate to functional gains in older adults recently discharged from the hospital. The findings support that many older adults with multiple chronic conditions, mild cognitive impairment, and severe functional limitation can meet frequency and intensity guidelines for cardiovascular exercise and frequency and repetition guidelines for resistance exercise. FITT parameter guidelines should be matched to a level that leads to physiological gains and should take into consideration the complex needs of older adults transitioning from hospital-to-home.

## Supplemental Material

Supplemental Material - Description and Functional Benefits of Meeting Frequency, Intensity, and Time of Resistance and Cardiovascular Exercises: A Study of Older Adults in a Community-Based, Slow-Stream Rehabilitation, Hospital-to-Home Transition Program Click here for additional data file. Supplemental Material for Description and Functional Benefits of Meeting Frequency, Intensity, and Time of Resistance and Cardiovascular Exercises: A Study of Older Adults in a Community-Based, Slow-Stream Rehabilitation, Hospital-to-Home Transition Program by Melody Maximos, Paul Stratford, Ada Tang, and Vanina Dal Bello-Haas in Gerontology and Geriatric Medicine.
