# Supplementary material for: Description and Functional Benefits of Meeting Frequency, Intensity, and Time of Resistance and Cardiovascular Exercises: A Study of Older Adults in a Community-Based, Slow-Stream Rehabilitation, Hospital-to-Home Transition Program
Source: Gerontol Geriatr Med. 2022 May 20;8:23337214221096303. doi: 10.1177/23337214221096303 (PMC9125058; doi:10.1177/23337214221096303)
Supplement: Supplemental Material - Description and Functional Benefits of Meeting Frequency, Intensity, and Time of Resistance and Cardiovascular Exercises: A Study of Older Adults in a Community-Based, Slow-Stream Rehabilitation, Hospital-to-Home Transition Program [file sj-pdf-1-ggm-10.1177_23337214221096303.pdf]

Running head: EXERCISES COMPLETED IN COMMUNITY-BASED, SSR PROGRAM

Supplementary Material

Table 1A

Frequency, Intensity Time and Type (FITT) Parameters of Exercises Completed by Participant

Exercise Type	FIT Parameter	Mean (SD)	Median	Mode	Minimum, Maximum	Number of Participants (%)
Cardiovascular	Frequency (days/week)	3.4 (1.5)	3.2	5	0, 5	12 (18.7%)-once a week<br>14 (21.9%) - 2 times/week<br>11(17.2%)- 3 times/week<br>8 (12.5%) - 4 times/week<br>19 (29.7%) - 5 times/week
	Intensity (CR-10 Borg ® RPE scale)	5.0 (1.7)	4.2	5-6	1, 9	16 (25.0%) - RPE <3 (light)<br>18 (28.1%) - RPE 3-4 (Moderate)

For Peer Review

						23 (35.9%) - RPE 5-6 (strong/hard)<br>6 (9.4%) - RPE 7-8 (very strong)<br>1 (1.6%) - RPE 9-10 (Maximal)[a]
	Time (minutes)	14.8 (3.7)	14.2	20	5, 30	20 (31.3%) - <10 minutes<br>21 (32.8%) - 10 -19 minutes<br>22 (34.3 %) - 20-25 minutes<br>1 (1.6%) - 30 minutes
Resistance - lower body	Frequency (days/week)	3 (1.8)	3.2	5	0, 5	8 (12.5%) - did not do<br>11(17.2%) - once a week<br>14 (21.9%)- 2 times/week<br>10 (15.6%) -3 times/week

For Peer Review

						4 (6.2%) – 4 times/week<br>17 (26.6%) - 5 times/week
	Intensity (CR-10 Borg ® RPE scale)	5.6 (1.7)	5.5	5-6	2, 10	8 (12.5%) - did not do<br>8 (12.5%)- RPE <3 (light)<br>18 (28.1%) - RPE 3-4 (Moderate)<br>23 (35.9%)- RPE 5-6 (strong/hard)<br>6 (9.4%)- RPE 7-8 (very strong)<br>1 (1.6%) - RPE 9-10 (Maximal)[a]
	Time (number of	16.5 (7.2)	20	20	0, 25	8 (12.5%) - no repetitions<br>5 (7.8 %) < 8 repetitions<br>51 (79.6%)- 20 to 25 repetitions

For Peer Review

	repetitions per exercise)					
Resistance - Upper body	Frequency (days/week)	2.8 (1.9)	2	5	0, 5	12 (18.8%) - did not do<br>11 (17. 2%) -once a week<br>9 (14.1%) - 2 times/week<br>9 (14.1%) - 3 times/week<br>8 (12.5%) - 4times/week<br>15 (23.4%) - 5 times
	Intensity (CR-10 Borg® RPE scale)	4.7 (1.4)	4.7	5-6	2, 9	12 (18.8%) - did not do<br>6 (9.3%) - RPE <3 (light)<br>21 (32.8%) - RPE 3-4 (Moderate)<br>23 (35.9%) - RPE 5-6 (strong/hard)

For Peer Review

						1 (1.6%) - RPE 7-8 (very strong)<br>1 (1.6% - RPE 9-10 (Maximal)[a]
	Time (number of repetitions per exercise)	14.9 (8.3)	20	15	0, 20	12 (18.8%)- no repetitions<br>5 (7.8%) < 8 repetitions<br>47 (73.4%) - 15-20 repetitions

Note: [a]One participant

For Peer Review
